# Supplementary material for: Factors Associated with Injury Rate and Pregnancy Success in Rhesus Macaques
Source: Biology (Basel). 2022 Jun 28;11(7):979. doi: 10.3390/biology11070979 (PMC9311521; doi:10.3390/biology11070979)
Supplement: Supplementary file 1 [file biology-11-00979-s001.zip › biology-1752983-Figure S1 and Table S1.pdf]

Supplementary Data, Figure S1 and Table S1

The supplementary data consists of two csv files containing the data used in the publication, the three r scripts used to generate the generalised mixed effect models in Results sections 3.1, 3.4 and 3.5 and the supplementary **F**igure S1 **and Table S1**.

Results Section 3.1 (Distribution of Injuries by Age, Sex and Group Type)

Data: Data_Results_Section_3_1.csv
R Script: RCode_Results_Section_3_1.R
Random Effect Distribution: Figure S1A (Individual IDs) and S1B (Group IDs)

Results Section 3.4 (Injury Rate in Breeding Females)

Data: Data_Results_Section_3_4.csv
R Script: RCode_Results_Section_3_4.R
Random Effect Distribution: Figure S1C (Individual IDs) and S1D (Group IDs)

Results Section 3.5 (Productivity of Breeding Females)

Data: Data_Results_Section_3_4.csv (same csv file as in previous section)
R Script: RCode_Results_Section_3_5.R
Random Effect Distribution: Figure S1E (Individual IDs) and S1F (Group IDs)

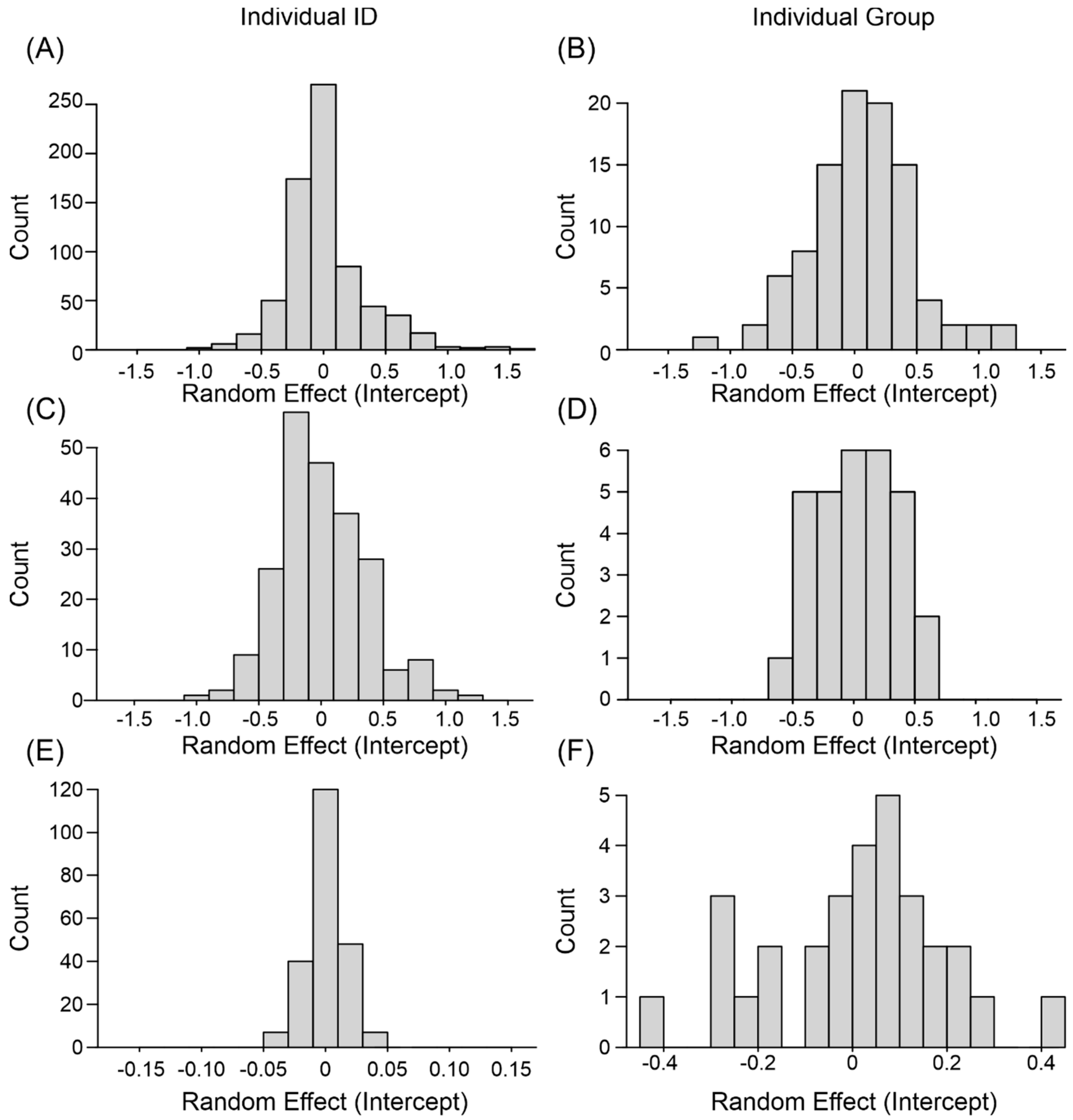

Figure S1. Distribution of random effects. (A) Individual ID and model in Results section 3.1. (B) Group ID and model in Results section 3.1. (C) Individual ID and model in Results section 3.4. (C) Group ID and model in Results section 3.4. (E) Individual ID and model in Results section 3.5. (F) Group ID and model in Results section 3.5.

As discussed in the main text we were unable to include dominance rank in the main injury model for the breeding females as we didn't have reliable rank information for many of the females. We have run the model on a subset of the females where we did have an estimate of rank (broadly classified into high, mid and low ranking, 125 of the original 222 females). The model was:

Injury ~ AgeC + Oldest_FemaleC + UnrelatedFemales + Season + Group_NewMale + Pregnant + Infant + Female_Rank+Group_MaleAgeC*FemalesC+(1|ID_Ind)+(1|Unique_Group_ID)

Table S1. Model Including Dominance Rank.

Predictor Variable	Estimate	SE	Z	P
Intercept	−2.48	0.191	−13.02	$< 2 \times 10^{-16}$ ***
**Fixed terms**				

Age	0.09	0.016	5.47	4.63×10^{-8} ***
Oldest Female	−0.01	0.017	−0.46	0.6487
Unrelated Females: True	−0.05	0.171	0.31	0.7539
Season: Mar-May [1]	−0.04	0.097	−0.44	0.6570
Season: Jun-Aug [1]	−0.05	0.096	−0.55	0.5806
Season: Sep-Nov [1]	0.15	0.091	1.63	0.1035
New Male: Introduction [1]	1.18	0.171	6.87	6.44×10^{-12} ***
New Male: First 3 Months [1]	0.51	0.122	4.20	2.72×10^{-5} ***
Is Pregnant: True	0.11	0.082	1.36	0.1748
Has Infant: True	0.05	0.076	0.64	0.5195
Dominance Rank: Mid	0.05	0.134	0.34	0.7340
Dominance Rank: Low	0.22	0.146	1.51	0.1309
Age of Breeding Male	−0.07	0.010	−7.30	2.86×10^{-13} ***
Number of Females	−0.08	0.025	−3.24	0.0012 **
**Interactions**				
Age of Male x No of Females [2]	−0.018	0.004	−4.367	1.26×10^{-5} ***
**Random terms**	Variance	N		
Animal ID [3]	0.168	125		
Unique Group ID [3]	0.094	25		

[1] The reference category is the established groups. [2] Interaction between the age of the breeding male and the number of breeding females. [3] Random intercepts were fitted for animal ID and unique group ID. ** Significance $p < 0.01$, *** Significance $p < 0.001$.

Including rank had no effect of the outcome of the other factors – the main significant factors are still the age of the female, the introduction of a new male, the age of the breeding male, the group size and the interaction between male age and group size.
